# Autoserum: An Optimal Supplement for Bone Marrow Mesenchymal Stem Cells of Liver-Injured Rats

**DOI:** 10.1155/2015/459580

**Published:** 2015-05-24

**Authors:** Qinglin Zhang, Xun Sun, Jianxun Ding, Ping He, Yujia Liu, Hongjing Cheng, Changlin Zhou, Xiangwei Meng

**Affiliations:** ^1^Department of Gastroenterology, The First Hospital of Jilin University, Changchun 130021, China; ^2^Department of Pathology, The First Hospital of Jilin University, Changchun 130021, China; ^3^Key Laboratory of Polymer Ecomaterials, Changchun Institute of Applied Chemistry, Chinese Academy of Sciences, Changchun 130022, China; ^4^Department of Endocrinology, The First Hospital of Jilin University, Changchun 130021, China

## Abstract

Mesenchymal stem cells (MSCs) are an attractive source for the clinical cell therapy of liver injury. Although the use of adult serum, platelet lysate, or cord blood serum solves some of the problems caused by fetal bovine serum (FBS), the allogeneic immune response, contamination, and donor-to-donor and donor-to-receptor differences still obstruct the application of MSCs. In this study, the influences of autoserum from liver-injured rats (LIRs) and allogeneic serum from healthy rats on the isolation and culture of bone marrow MSCs (BMSCs) were examined and compared to FBS. The results showed that BMSCs cultured with autoserum or allogeneic serum exhibited better MSC-specific morphology, lower rate of cell senescent, and higher proliferation kinetics than those with FBS. In addition, autoserum promoted the osteogenic differentiation potential of BMSCs as allogeneic serum did. Although there were no significant differences in proliferation activity, immunophenotypic characterization, and differentiation potential between BMSCs cultured with autoserum and those with allogeneic serum, the potential adverse immunological reactions in patients with allogeneic material transplantation must be considered. We therefore believe that the autoserum from liver-injured patients may be a better choice for MSC expansion to meet the needs of liver injury therapy.

## 1. Introduction

Mesenchymal stem cells (MSCs) were first described by Friedenstein and colleagues in 1987 [1]. MSCs are characterized by their multilineage differentiation, self-renewal, anti-inflammatory, immunosuppressive functions, and migration potential [2–4]. For potential treatments, MSCs can differentiate into osteocytes, chondrocytes, cardiomyocytes, endothelial cells, lung epithelial cells, hepatocytes, and neurons and promote the repair of damaged tissues [5, 6]. Moreover, various factors, such as interleukin-6 (IL-6), interleukin-10 (IL-10), macrophage colony-stimulating factor (M-CSF), prostaglandin E2 (PGE2), and hepatocyte growth factor (HGF), secreted by MSCs can stimulate the proliferation and differentiation of endogenous tissue progenitors and decrease inflammatory and immune reactions [5].

To meet the requirements of various treatments, the acquisition of high-quality MSC is indispensable. Taking acute myocardial infarction, for example, Jin and colleagues found that the first generation of MSCs had better therapeutic efficacy in mouse model compared to the fifth passage ones [7]. Although researchers can expand enough MSCs at a good manufacturing practice (GMP) grade, the inconsistent stem cell potency, poor cell engraftment, and age/disease-related host tissue impairment are inevitably encountered in MSC therapy [8]. It is therefore necessary to develop an appropriate culture system* in vitro* that allows for the stable expansion of stem cells for clinical application.

Serum is an indispensable part of cell culture medium, and fetal bovine serum (FBS) is the most widely used serum for MSC culture. However, FBS, as an animal serum, can be a potential source of microbial contaminants, such as fungi, bacteria, mycoplasma, viruses, or prions, and different batches of serum display quantitative and qualitative variations in their composition [9]. In addition, the adverse immunological reactions induced by heterologous serum are a hurdle that should be taken into consideration [10, 11]. Several novel culture supplements, such as adult serum, platelet lysate, umbilical cord extract, and cord blood serum, have been tested with ideal results [12–16]. In order to avoid cross-contamination and the immune response induced by allogeneic transplantation, we tried to apply autologous supplements to culture MSCs for liver injury therapy.

Platelet lysate and cord blood serum contain higher contents of growth factors than FBS or adult serum [16–19]. These growth factors may play an important role in maintaining the properties of MSCs. Similarly, the contents of some growth factors, such as insulin-like growth factor-1 (IGF-1) and platelet-derived growth factor BB (PDGF-BB), are higher in the blood of patients suffering from liver injury than in the blood of healthy people [20, 21]. The levels of basic fibroblast growth factor (bFGF), epidermal growth factor (EGF), and HGF are known to be higher in the damaged liver tissue [22–24]. These factors may also affect MSC proliferation and properties.

In this study, the autoserum and bone marrow mesenchymal stem cells (BMSCs) from the same liver-injured rat (LIR) were used to evaluate the feasibility of using autoserum as a replacement for FBS in BMSCs culture. Allogeneic serum from healthy rats was used as a control to demonstrate the superiority of autoserum in BMSCs culture.

## 2. Materials and Methods

### 2.1. Animals

Adult male Wistar rats weighing 200–220 g were purchased from the Animal Center of Jilin University. All rats were housed in room maintained at constant temperature and humidity with a 12/12 h light/dark cycle according to the guidelines established by Jilin University. All experimental procedures were approved by the Supervisory Committee of Jilin University Animal Council.

### 2.2. Preparations of Autoserum and Allogeneic Serum

Liver injury in rats was induced by intraperitoneal administration of a single dose of 0.5 mL carbon tetrachloride (CCl_4_) per kg body weight [25]. On the 4th day after CCl_4_ administration, the anesthetized rats (*n* = 16) were sequentially disinfected with 75% alcohol and povidone-iodine. Rat blood was drawn through the inferior vena cava, and the rats were then sacrificed. Liver tissues of each rat were clipped after the sacrifice and stored in formalin for the follow-up experiments. The femora and tibiae of LIRs were separated, and the muscle tissues were dropped. The whole blood samples were injected into 15 mL sterile tubes in the absence of anticoagulant and incubated at 37°C for 30 min or until the occurrence of clotting. The serum was separated by centrifugation at 3,000 g for 10 min. Subsequently, the supernatant was filtered using a 0.44 *μ*m filter (BD Biosciences, San Jose, CA, USA), transferred into new 15 mL sterile tubes, and stored at 4°C. Healthy rats (*n* = 16) were subject to the same procedure to prepare allogeneic serum.

### 2.3. Isolations and Cultures of BMSCs from LIRs

BMSCs were obtained as described previously [26]. First, the bone marrow of femora and tibiae was washed out with phosphate-buffered saline (PBS). Cells from the same rat were split into three equal subfractions, centrifuged at 1,500 g for 5 min in 15 mL sterile tubes, and resuspended in 2.0 mL of Dulbecco's modified Eagle's medium (DMEM): Nutrient Mixture F-12 (DMEM/F-12; Life Technologies, Inc., Gaithersburg, MD, USA) supplemented with 10% (V/V) autoserum (AuSM), allogeneic serum (AlSM), or FBS (HyClone, Australia; FSM). The resuspended cells were inoculated into 6-well plates (Corning, USA) containing different media and incubated at 37°C in a humidified atmosphere containing 95% air and 5% (V/V) carbon dioxide (CO_2_). All nonadherent cells were removed after 72 h. When these primary cultures reaching ~70% confluence, the cells were subcultured with the same medium used in primary cultures.

### 2.4. Assessments of Senescence-Associated *β*-Galactosidase (SA-*β*-gal) Staining

Passage 4 (P4) BMSCs from different medium was seeded into 6-well plates with a density of 1 × 10^5^ cells/well. After 24-h culture, the cells were stained by SA-*β*-gal to detect cell senescence using the SA-*β*-gal kit (Beyotime, Beijing, China) as per the instructions of the manufacturer. The senescent cells were observed with an optical microscope and counted in three random fields of vision.

### 2.5. Proliferation Kinetics

The expansion of BMSCs using different supplements were counted and passaged at a confluence of 70–80%. At each passage, the population doubling rate (PDR) and population doubling time (PDT) were determined according to the following equation: PDR = [lg(*N*_*h*_) − lg(*N*_1_)]/lg(2) and PDT = *T*lg(2)/[lg(*N*_*h*_) − lg(*N*_1_)], where *N*_1_ is the planted cell number, *N*_*h*_ is the cell number at harvest, and *T* is the time in culture. To calculate the cumulative population doubling (CPD) rate, the PDR for each passage was determined and then added to the previous passages.

At the same time, a growth curve of BMSCs expanded with the above three different supplements was started at P4. Cells were planted in 24-well plates at 1 × 10^4^ cells/cm^2^, and three wells were trypsinized (Trypsin (0.25%)/EDTA; Life Technologies, Inc., Gaithersburg, MD, USA) and counted every 24 h for 7 days.

### 2.6. Immunophenotypic Analyses

Immunophenotypic analyses were performed for BMSCs from different culture media at P4 following a previously published protocol [27]. After incubation, cells were treated with antibodies, including anti-rat CD44, CD73, CD90, CD34, and CD45 (eBioscience, San Diego, CA, USA). At last, FACS Calibur flow cytometer (BD Biosciences, San Jose, CA, USA) equipped with Cell Quest Pro Analysis software (BD Biosciences) was employed to analyze the phenotypes.

### 2.7. *In Vitro* Differentiation Assays

To test the differentiation into adipogenic cells or osteogenic cells, P4 BMSCs were treated as described previously [27]. After 3-week induction culture in an adipogenic or osteogenic medium, cells were stained with Oil Red O (ORO) or Alizarin Red S (ARS) correspondingly. The optical densities of ARS and ORO were measured at 405 and 520 nm, respectively [28].

### 2.8. Statistical Analyses

All data were expressed as mean ± standard deviation (SD). Multiple comparisons between the two groups were analyzed by the Kruskal-Wallis *H* nonparametric test. All statistical analyses were performed by the SPSS software suite (V19.0 for Windows; SPSS, Inc., Chicago, IL, USA). A two-sided *P* value of <0.05 was considered statistically significant.

## 3. Results

### 3.1. Verification of CCl_4_-Induced Acute Liver Injury

**Figure 1:**
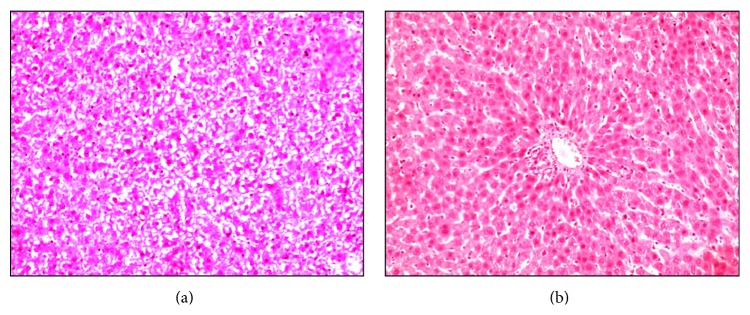
Histological microimages of liver tissues from (a) rat model of acute liver failure and (b) healthy control rat stained with H&E. Magnification: ×200.

Hematoxylin and eosin (H&E) staining on the liver tissue section was employed to verify the induction of acute liver injury in rat. Extensive vacuolar, fatty, and hydropic degeneration were detected in the liver of rat that received a dose of 0.5 mL CCl_4_ per kg body weight (Figure 1(a)). These features were accompanied by vacuole-shaped cytoplasm, karyorrhexis, pyknosis, or karyolysis in cell nucleus with respect to the histopathological morphology of liver tissue in healthy rat (Figure 1(b)). These observations were consistent with the histological characteristics of acute liver injury.

### 3.2. Morphologies and Growth Trends

**Figure 2:**
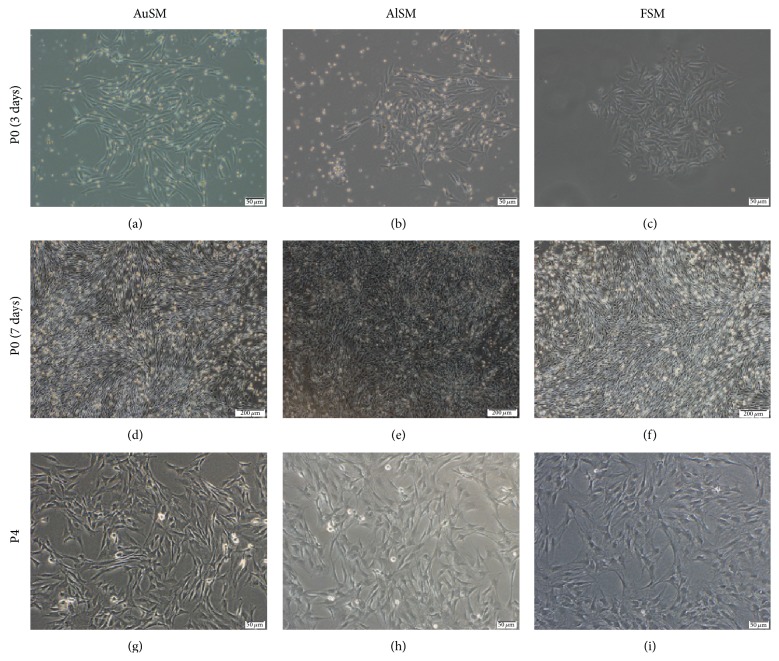
Morphologies of primary and secondary (P4) cultures of BMSCs cultured in (a, d, and g) AuSM, (b, e, and h) AlSM, and (c, f, and i) FSM. Scale bars in (a), (b), (c), (g), (h), and (i) = 50 *μ*m, and scale bars in (d), (e), and (f) = 200 *μ*m.

In primary culture, a small amount of short spindle cells crept into a whorled architecture after being cultured for 3–5 days (Figures 2(a)–2(c)). At this point, there was no obvious difference among the different culture media. With the prolonged incubation time, the number and size of long spindle cells increased (Figures 2(d)–2(f)). Cells cultured with autoserum (Figure 2(e)) and allogeneic serum (Figure 2(d)) appeared smaller and more spindle shaped, while cells cultured with FBS showed larger cell size and more irregular morphology (Figure 2(f)).

We were able to successfully expand the cells with different media from P0 to P6. Starting at P4, the FSM-incubated BMSCs lost their spindle-like shape and showed a bigger, more flattened, and irregular morphology, whereas the AuSM-incubated and AlSM-incubated BMSCs retained their spindle-shaped morphology for longer time and did not show the same growth pattern as the FSM-incubated ones in P4 till the end of the experiment (Figures 2(g)–2(i)).

### 3.3. Senescence Analyses

**Figure 3:**
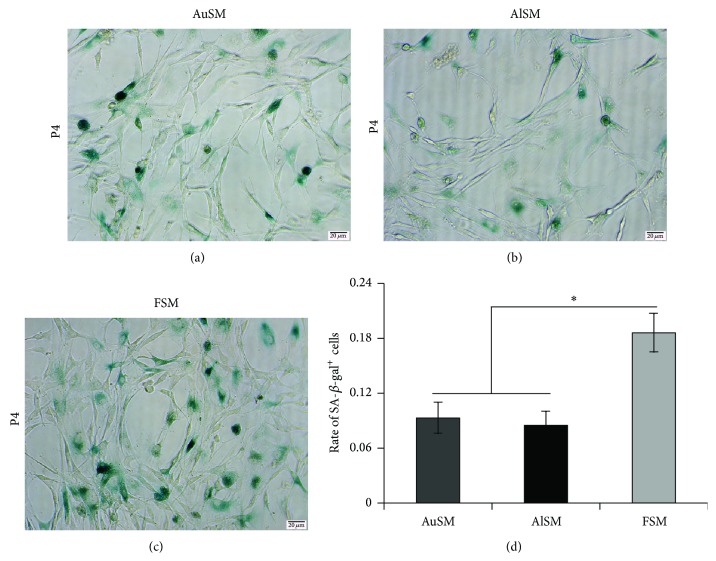
Senescence analyses of BMSCs cultured in AuSM, AlSM, and FSM. (a–c) SA-*β*-gal staining of BMSCs cultured in (a) AuSM, (b) AlSM, and (c) FSM. (d) Mean percentage of senescent cells in BMSCs from AuSM, AlSM, and FSM (*n* = 3). Scale bars = 20 *μ*m.

In order to detect whether different morphological changes were accompanied by varying extent cell senescence, SA-*β*-gal staining was employed for the detections of BMSCs at P4. There was obvious difference among BMSCs cultured in AuSM or AlSM versus FSM (Figures 3(a)–3(c)). The percentage of senescent cells in AuSM and AlSM was, respectively, 0.085 ± 0.015 and 0.093 ± 0.017, while that in FSM was 0.186 ± 0.021 (Figure 3(d)).

### 3.4. Proliferation Activities

**Figure 4:**
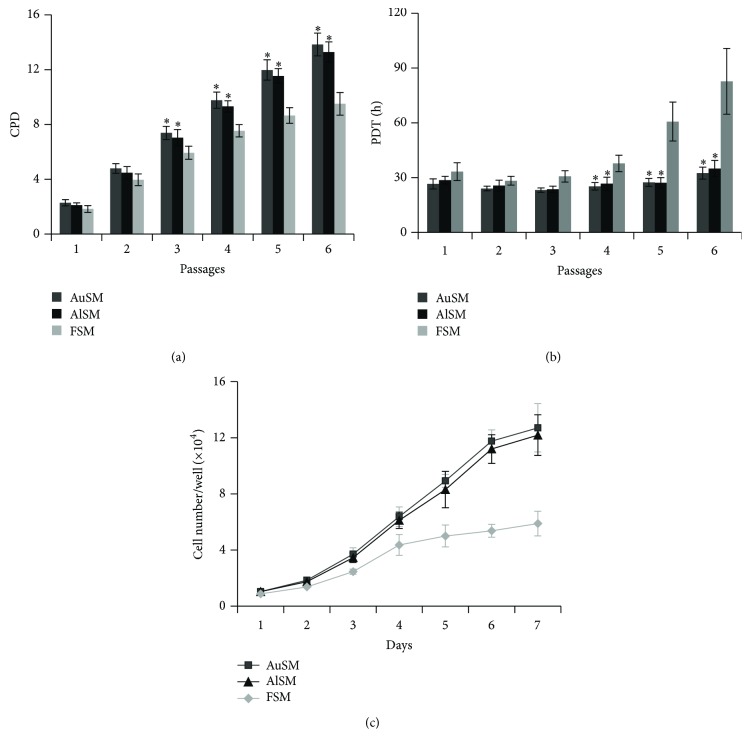
Proliferation of BMSCs cultured in AuSM, AlSM, and FSM. (a) Mean CPD for BMSCs cultivated in AuSM, AlSM, and FSM (*n* = 10). (b) Mean PDTs for BMSCs incubated in AuSM, AlSM, and FSM (*n* = 10). (c) Growth curves of BMSCs cultured in AuSM, AlSM, and FSM (*n* = 3). ^∗^Significantly different from BMSCs cultured in FSM (*P* < 0.05).

To examine the proliferation activity, the CPD and PDT of BMSCs cultured under different media were compared. CPD values were calculated for all passages to compare the effect of different supplements on the expansion capacity of MSCs. The data showed that CPD was significantly higher when BMSCs were cultured in AuSM or AlSM rather than that in FSM (Figure 4(a)). We also compared the PDT of different generations and found that the AuSM-incubated and AlSM-incubated BMSCs had signally shorter PDT from P2 to P5 (Figure 4(b)). These data suggested that BMSCs cultured in AuSM or AlSM showed increased proliferation potential. There were no significant differences between the AuSM-incubated and AlSM-incubated BMSCs.

To further clarify the influence of different culture media on cell proliferation, the growth curves of MSCs at P4 were started for different brands of FBS (*n* = 3; see Supplementary Figure S1 of the Supplementary Material available online at http://dx.doi.org/10.1155/2015/459580) and all culture conditions (*n* = 3; Figure 4(c)). The results of different brands of FBS indicated that there was no clear difference among BMSCs cultured with FBS from HyClone (Victoria, Australia), Gibco (USA), or BioInd (Israel). The data of different culture conditions showed that the AuSM-incubated and AlSM-incubated MSCs had a higher adhesion rate and a shorter latent phase at the first day. The AuSM-incubated and AlSM-incubated MSCs reached a final cell count of 12.71 ± 1.72 and 12.19 ± 1.45, respectively, on Day 7. In comparison, the FBS-incubated MSCs reached a cell count of only 5.89 ± 0.878 on Day 7. Thus, AuSM or AlSM had a high stimulatory effect on MSCs causing them to multiply about 12-fold within 7 days as opposed to the 6-fold expansion observed for FBS in the same time span. Considering that AuSM and AlSM flasks nearly reached confluence on Day 5, the proliferation of these cells might be even higher if supplied with a lower seeding density and a larger growth area.

### 3.5. Immunophenotypic Characterizations

**Figure 5:**
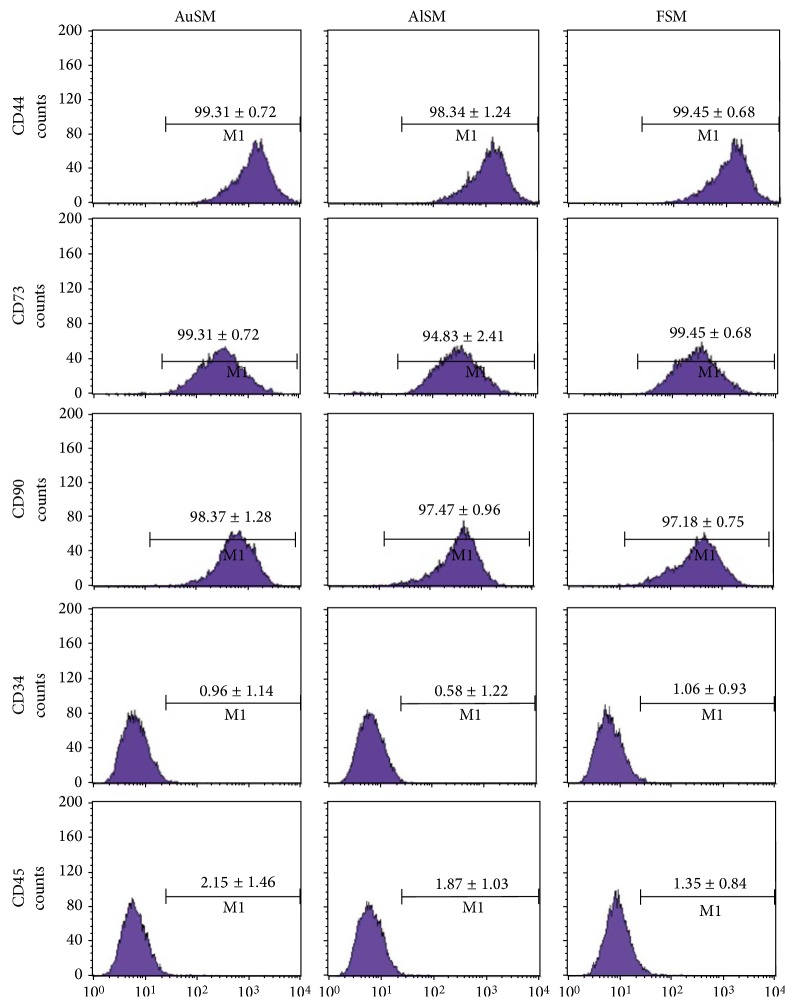
Flow cytometry analyses of surface markers expressed in BMSCs. BMSCs cultured in AuSM, AlSM, and FSM were positive for CD90, CD73, and CD44 but negative for CD31 and CD45 (*n* = 3). The expression of surface markers did not depend on culture conditions.

The expression of selected surface markers of CD44, CD73, CD90, CD34, and CD45 was evaluated using flow cytometry. BMSCs were cultured in AuSM, AlSM, or FSM for the total of three passages prior to analysis. The cells were positive for CD90, CD73, and CD44 and negative for CD34 and CD45 (Figure 5). There was no significant difference in the expression of surface markers depending on various culture conditions (*P* > 0.05).

### 3.6. Differentiation Potentials

**Figure 6:**
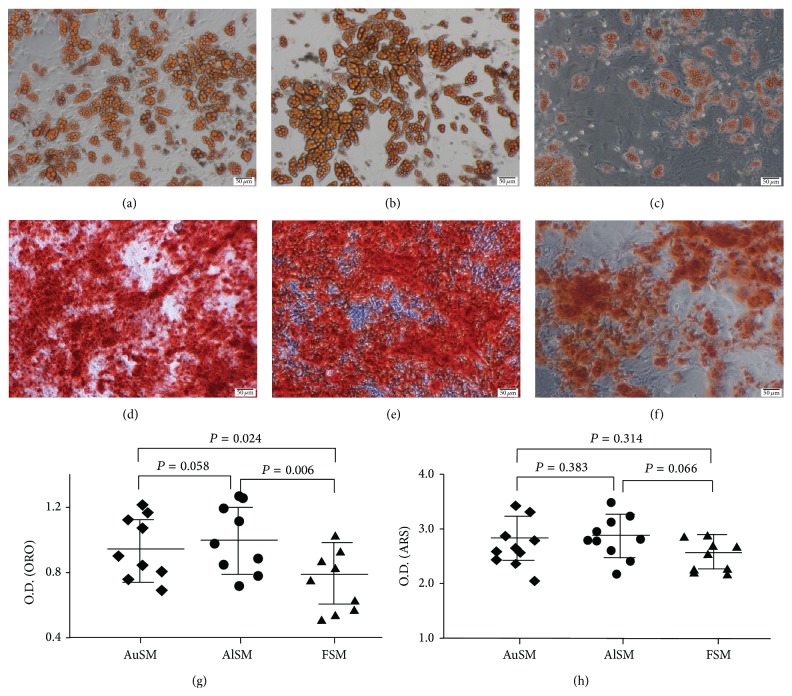
Differentiation potentials of BMSCs cultured in AuSM, AlSM, and FSM. (a–c) ORO staining of BMSCs cultured in (a) AuSM, (b) AlSM, and (c) FSM differentiated into adipocytes. (d–f) ARS staining of BMSCs cultured in (d) AuSM, (e) AlSM, and (f) FSM differentiated into osteocytes. (g) Quantification of oil droplets at 520 nm after ORO staining. (h) Quantification of mineralization at 405 nm after ARS staining. Scale bars = 50 *μ*m.

Differentiation potential is one of the most important characteristics of MSCs. The properties of MSCs can be assessed by assaying their potential to differentiate along the adipogenic and osteogenic lineages [29]. In this study, the adipogenic and osteogenic capacities of P4 BMSCs cultured in different media were therefore compared. Adipogenic differentiation was confirmed by the formation of lipid vacuoles that were stained by ORO (Figures 6(a)–6(c)), while osteogenic differentiation was indicated by the deposition of a mineralized matrix stained with ARS (Figures 6(d)–6(f)). The successful induction of differentiation from both media was observed in MSCs cultured in AuSM, AlSM, and FSM.

To determine whether the differentiation potential changed, the uptake of dyes was further quantified by a spectrophotometric analysis. The data showed that the AuSM-incubated and AlSM-incubated BMSCs could produce more lipid vacuoles matrix than the FSM-incubated ones (Figure 6(g)), while no significant difference was found among different cells from various conditions after being cultured in the osteogenic induction medium (Figure 6(h)). In addition, no significant difference was found in the adipogenic and osteogenic capacities of the BMSCs incubated in AuSM and AlSM.

## 4. Discussion

With the properties of multilineage differentiation potential, self-renewal, and factor secretion according to the environment, MSCs are ideal seed cells for many disease treatments, including myocardial infarction, brain and spinal cord injuries, and kidney damage [5, 7]. Because of organ shortage for liver transplantation and the absence of other effective treatments, MSC therapy has also become the new hope for liver diseases [6, 30]. However, most of MSCs are cultivated with FBS, which have some limitations associated with their clinical applications, such as prion and viral transmission, adverse immunological reactions, and cross contamination with xenogeneic components [31]. Some studies have tested human serum, plasma, platelet lysate, umbilical cord extract, and cord blood serum as the substitutes for FBS and have found that these substitutions can significantly stimulate the proliferation of MSCs [12–18]. Nonetheless, all these previous protocols used allogeneic materials, while the cells from medium according with the internal environment of patients may lead to better therapeutic efficacy. People in need of transplantation therapy are in diseased state, so we try to evaluate the effect of autoserum on the morphologies and behaviors of BMSCs in comparison to allogeneic serum and FBS.

Ma and colleagues have shown that MSCs expanded with cord blood serum showing fewer cytoplasmic processes and retaining their spindle-shaped morphology during long-term culture than those MSCs which expanded with FBS [16]. In this study, a rat model of acute liver injury was successfully established, and BMSCs were obtained by being cultured with different serums. As predicted, most of BMSCs cultivated with autoserum or allogeneic serum maintained the spindle shape for longer time than those cultivated with FBS.

Although BMSCs have the ability to regenerate themselves, senescence and spontaneous differentiation, which may lead to inconsistencies between study results, are inescapable facts in BMSCs expansion* in vitro* [32–35]. In addition, senescence may give rise to spontaneous malignant transformation and seriously affect the clinical applications of MSCs [36]. Many studies have verified that the medium types of MSCs expansion used considerably influence morphology, proliferation, passage number, clonogenicity, and differentiation potential [37, 38]. As confirmed by Dahl and coworkers, BMSCs expanded in autoserum tend to generate more consistent genomic backgrounds and DNA methylation patterns [39]. In order to detect the influence of serum on BMSC senescence, SA-*β*-gal staining was performed. The data showed that BMSCs cultured in AuSM and AlSM revealed lower percentage of senescent cells compared to that in FSM. The results, which were consistent with previous studies [39], suggested that autoserum and allogeneic serum might be better supplements for the stable culture of BMSCs.

Most clinical cell therapies require a large number of cells, that is, >2 × 10^6^ cells per kg body weight [18, 40], and it is hard to generate enough MSCs [41]. Researchers have long tried to find a safe and efficient protocol to expand MSCs, and it has been found that autologous human platelet lysate from less than 70 mL of whole blood can satisfy expansion need [42]. In this study, we have shown that both autoserum and allogeneic serum had a significantly higher proliferative effect on BMSCs than FBS, providing a 12-fold expansion within 7 days compared with a 6-fold expansion for FBS in the same time span. Moreover, BMSCs cultured in AuSM and AlSM revealed higher CPD and less PDT. All these facts made autoserum and allogeneic serum attractive candidates for MSC expansion in clinical settings. The large number of MSCs required for transplantation could be achieved in a short period of time. About 3-4 mL of autoserum can be obtained from a single rat, and with this serum, about 1.5 × 10^6^ BMSCs can be harvested. According to this model, 2 × 10^8^ BMSCs, which are enough for liver injury therapy, can be obtained from about 150.0 mL of autoserum.

Besides fibroblast-like morphology, specific phenotype and differentiation potential are the other two essential properties for BMSCs identification [29]. The data of this study showed that there was no significant different on phenotypic markers between BMSCs from various media. In addition, BMSCs cultured in AuSM and AlSM retained their differentiation capacity to osteogenic and adipogenic lineages as the FSM-incubated ones. The results indicated that the culture with autoserum and allogeneic serum did not change the essence of traditional isolation and culture methods of BMSCs [43].

In this study, both autoserum and allogeneic serum enhanced the adipogenic differentiation of BMSCs as umbilical cord blood serum did, while they did not affect osteogenic differentiation [28]. Peng and colleagues found that the differentiation potential of MSCs was closely related to cell size and density, and smaller MSCs and higher cell density could lead to higher adipogenic differentiation [44]. As shown in our cell growth studies, BMSCs cultured in AuSM and AlSM had smaller cell size and higher cell density as well as higher adipogenic differentiation. So we believe that the BMSCs cultured with autoserum and allogeneic serum have better stem cell condition and are more suitable for disease treatment.

Although there was no significant difference between autoserum and allogeneic serum in proliferation activity, immunophenotypic characterization, and differentiation potential, the potential adverse immunological reactions in patients and allogeneic material transplantation must be considered. Moreover, autoserum culture system is more in line with the concept of autologous transplantation. So the autoserum from liver-injured patients may be a better choice for BMSC therapy than allogeneic serum.

## 5. Conclusions

As allogeneic serum, autoserum could promote proliferation and reduce senescence of BMSCs. Moreover, BMSCs cultured in AuSM maintained better stem cell morphology and exhibited higher potential for adipogenic differentiation, comparing to that in FSM. In addition, considering the potential adverse immunological reactions of allogeneic materials and the concept of autologous transplantation, autoserum from liver-injured body seems to be the optimal supplement for liver injury therapy.

## Supplementary Material

The supplementary materials contain the detection methods and results of the proliferation activities of BMSCs, which were cultured in various media supplemented with different brands of FBS, including HyClone (Victoria, Australia), Gibco (USA), and BioInd (Israel).
