# Supplementary material for: Autoserum: An Optimal Supplement for Bone Marrow Mesenchymal Stem Cells of Liver-Injured Rats
Source: Stem Cells Int. 2015 May 24;2015:459580. doi: 10.1155/2015/459580 (PMC4458300; doi:10.1155/2015/459580)
Supplement: Supplementary file 1 — The supplementary materials contain the detection methods and results of the proliferation activities of BMSCs, which were cultured in various media supplemented with different brands of FBS, including HyClone (Victoria, Australia), Gibco (USA), and BioInd (Israel). [file 459580.f1.pdf]

Supplementary Materials

BMSCs Cultured with Different Brands of FBS

After being isolated from rat (*n* = 3), the BMSCs were cultured in various media supplemented with different brands of FBS, including HyClone (Victoria, Australia), Gibco (USA), and BioInd (Israel). The growth curves of BMSCs expanded using the three different brands of FBS were started at passage 4 (P4). Cells were plated in 24-well plates at 1×10^4 cells/cm^2, and three wells were trypsinized (Trypsin (0.25%)/EDTA; Life Technologies, Inc., Gaithersburg, MD, USA) and counted every 24 h for 7 days.

Supplementary Figure S1: To detect the proliferation activity of BMSCs cultured in those three brands of FBS, the growth cures of BMSCs was carried out. The results showed that there was no clearly different cell counts of 5.67 ± 0.726, 6.14 ± 0.568 and 5.41 ± 0.878 on Day 7 between HyClone, Gibco, and BioInd groups.

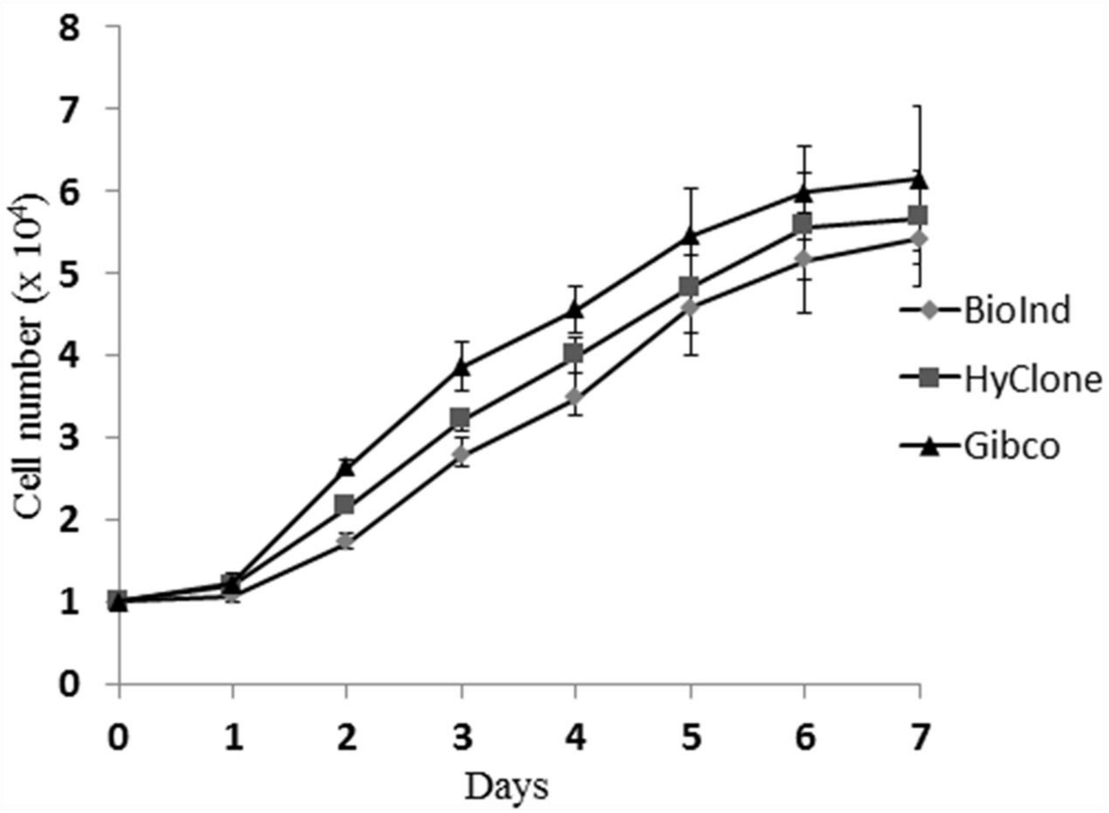

FIGURE S1

FIGURE S1. The growth cures of BMSCs cultured with different brands of FBS from HyClone (Victoria, Australia), Gibco (USA), and BioInd (Israel).
